# Relational nonlinear almost contractions of Lakshmikantham-Ćirić type and applications to boundary value problems

**DOI:** 10.1371/journal.pone.0356463

**Published:** 2026-08-31

**Authors:** Doaa Filali, Amal F. Alharbi, Mohammed Zayed Alruwaytie, Abdulaziz Abbas Alshammari, Faizan Ahmad Khan, Adel Alatawi

**Affiliations:** 1 Department of Mathematical Science, College of Sciences, Princess Nourah Bint Abdulrahman University, Riyadh, Saudi Arabia; 2 Department of Mathematics, King Abdulaziz University, Jeddah, Saudi Arabia; 3 Department of Basic Sciences, College of Science and Theoretical Studies, Saudi Electronic University, Riyadh, Saudi Arabia; 4 Department of Mathematics, University of Tabuk, Tabuk, Saudi Arabia; Birla Institute of Technology & Science Pilani - Hyderabad Campus, INDIA

## Abstract

In this paper, we explore specific fixed point outcomes under a Lakshmikantham-Ćirić type nonlinear description of almost contractions in a metric space with a locally ℏ-transitive binary relation. Numerous previous insights are expanded, developed, improved, and consolidated in the outcomes reported herein. To demonstrate the reliability of our results, a few instances are supplied. Our outcomes are deployed to evaluate the accuracy of the unique solution of a boundary value problem.

## Introduction

In 2004, Berinde [1] laid out a novel abstraction of BCP adopting the notion of almost contraction in 2004.

**Definition 1.** *[*1*] A self-mapping* ℏ *in a MS* (𝐕,ϱ) *is termed as an almost contraction if* ∃ 0<𝑐<1 *and* 𝑎≥0 *that enjoy*


$$\begin{array}{c} \varrho (\hbar v,\hbar w)\leq 𝑐\cdot \varrho (v,w)+𝑎\cdot \varrho (v,\hbar w),\;\forall \;v,w\in 𝐕. \end{array}$$

The preceding inequality and the subsequent one are identical because of the symmetry of ϱ:


$$\begin{array}{c} \varrho (\hbar v,\hbar w)\leq \text{c}\cdot \varrho (v,w)+\text{a}\cdot \varrho (w,\hbar v),\;\forall \;v,w\in 𝐕. \end{array}$$

**Theorem 1.** *[*1*] An almost contraction in a complete MS admits a fixed point.*

The class of almost contractions incorporates the maps defined by Banach, Chatterjea [2], Kannan [3], Zamfirescu [4], and a subclass of quasi-contractions due to Ćirić [5]. An almost contraction does not own a unique fixed. But, Picard’s recurrence sequence converges at a fixed point. A lot of investigators were developed the concept of almost contractions; e.g., see, Berinde and Păcurar [6], Ćirić et al. [7], Altun and Acar [8], and Aydi et al. [9] for some further information. Babu et al. [10] outlined a very reduced family of almost contractions in an effort to create a uniqueness theorem.

**Theorem 2.** *[*10*] Let* ℏ *be a self-mapping in a complete MS* (𝐕,ϱ)*. If* ∃ 0<𝑐<1 *and* 𝑎≥0 *with*


$$\begin{array}{c} \varrho (\hbar v,\hbar w)\leq 𝑐\cdot \varrho (v,w)+𝑎\cdot \min\{\varrho (v,\hbar w),\varrho (w,\hbar v),\varrho (v,\hbar v),\varrho (w,\hbar w)\},\;\forall \;v,w\in 𝐕, \end{array}$$

*then* ℏ *encompasses a unique fixed point.*

An intriguing and easily identifiable version of the BCP was reported by Alam and Imdad [11], in which MS occupies a BR that is revealed by the map. In the interim, Alam and Imdad [12] detected coincidence theorems in relational MS. Relation-theoretic contraction principle [11] is solidified and strengthened by lots of investigators incorporating different contractive-conditions, e.g., [13–24]. It can be noticed that such outcomes also improve the corresponding metrical fixed point theorems over digraphs (cf. [25–27]). All of such outcomes derive from their classical fixed point alternatives over universal BR. Because relational contractions are attributed to elements that continue to be associated by BR, they are far more extensive than Banach contractions. These outcomes are meant to be executed in scenarios where the conventional fixed point results are inappropriate, e.g., typical nonlinear integral equations, nonlinear matrix equations, and BVP, owing to their exclusive character.

Influenced by Browder [28] and Boyd and Wong [29], Lakshmikantham and Ćirić [30] endorsed the aforementioned collection of functions:


$$\begin{array}{c} \Theta =\left\{\theta :{\mathbb{R}}^{+}\to {\mathbb{R}}^{+}:\theta (\text{t})<\text{t},\;\forall \;\text{t}\in {\mathbb{R}}^{+}⧵\{0\}\;and\;\lim_{r\to {\text{t}}^{+}}\theta (\text{r})<\text{t},\;\forall \;\text{t}\in {\mathbb{R}}^{+}⧵\{0\}\right\}. \end{array}$$

Alfuraidan et al. [25] and Turinici [31], pursuant to the impact of Berinde [1], encountered the aforementioned collection of functions:


$$\begin{array}{c} ℳ=\{\mu :{\mathbb{R}}^{+}\to {\mathbb{R}}^{+}:\lim_{\text{r}\to 0^{+}}\mu (\text{r})=\mu (0)=0\}. \end{array}$$

This research examines the following nonlinear modification of the contraction condition that comprises Theorem 2 along a BR:


$$\begin{array}{c} \varrho (\hbar v,\hbar w)\leq \theta (\varrho (v,w))+\min\{\mu (\varrho (\text{v},\hbar w)),\mu (\varrho (w,\hbar v)),\mu (\varrho (\text{v},\hbar v)),\mu (\varrho (w,\hbar w))\}, \end{array}$$ (1)

whereas θ∈Θ and μ∈ℳ. We analyze fixed point theorems with regard to inequality (1) in the context of relational MS. This indicates that our main findings retain relatively vital than those of Babu et al. [10], Lakshmikantham and Ćirić [30], Turinici [31], Alfuraidan et al. [25], and similar others. We deliver a number of compelling instances that rationalize our findings. To highlight the breadth of our findings’ practical importance, we estimate a unique solution for a BVP in conjunction with certain other speculations.

## Relation-theoretic notions

A subset of ${𝐕}^{2}$ is termed as a BR ς on a set 𝐕. In the following, the ambient set is denoted by 𝐕, a metric on 𝐕 by ϱ, a BR on 𝐕 by ς, and a map by ℏ:𝐕→𝐕. We say that:

**Definition 2.** *[*11*]* 𝑣,w∈𝐕 *remain* ς*-comparative, indicated by* [𝑣,w]∈ς*, if*


$$\begin{array}{c} (𝑣,w)\in \varsigma \;\text{or}\;(w,𝑣)\in \varsigma . \end{array}$$

**Definition 3.** *[*32*]* $\varsigma^{-1}:=\{(𝑣,w)\in {𝐕}^{2}:(w,𝑣)\in \varsigma \}$ *retains inverse of* ς*.*

**Definition 4.** *[*32*]* $\varsigma^{s}:=\varsigma \cup \varsigma^{-1}$ *serves as symmetric closure of* ς*.*

**Proposition 1.** [11] $(𝑣,w)\in \varsigma^{s}\Leftrightarrow [𝑣,w]\in \varsigma .$

**Definition 5.** *[*11*]* ς *is* ℏ*-closed when*


$$\begin{array}{c} (𝑣,w)\in \varsigma ⟹(\hbar 𝑣,\hbar w)\in \varsigma . \end{array}$$

**Proposition 2.** *[*24*]* ς *is* $\hbar^{\text{n}}$*-closed for any* n∈ℕ*, when* ς *is* ℏ*-closed.*

**Definition 6.** *[*33*] A set* 𝐑⊆𝐕 *is* ς*-directed if for each* 𝑣,w∈𝐑, ∃ 𝑣∈𝐕 *that verifies* (𝑣,𝑣)∈ς *and* (w,𝑣)∈ς.

**Definition 7.** *[*11*] A sequence* $\{{𝑣}_{\text{n}}\}\subset 𝐕$ *is* ς*-preserving if* $({𝑣}_{\text{n}},{𝑣}_{\text{n}+1})\in \varsigma$, *∀* $\text{n}\in {\mathbb{N}}_{0}$.

**Definition 8.** *[*12*]* ℏ *is* ς*-continuous when for all* v∈𝐕 *and for each* ς*-preserving sequence* $\{v_{\text{n}}\}\subset 𝐕$ *verifying* $v_{\text{n}}\overset{\varrho }{⟶}v$*, we attain*


$$\begin{array}{c} \hbar (v_{\text{n}})\overset{\varrho }{⟶}\hbar (v). \end{array}$$

**Definition 9.** *[*12*]* (𝐕,ϱ) *remains* ς*-complete MS when every* ς*-preserving Cauchy sequence converges.*

**Definition 10.** *[*32*] For a subset* 𝐒⊆𝐕*, a BR* $\varsigma {|}_{𝐒}:=\varsigma \cap {𝐒}^{2}$ *retains the restriction of* ς *on* 𝐒*.*

**Definition 11.** *[*24*]* ς *retains locally* ℏ*-transitive when for each* ς*-preserving sequence* $\left\{v_{\text{n}}\}\subset \hbar (𝐕\right)$ *(with range* $𝐕=\{v_{\text{n}}:\text{n}\in \mathbb{N}\})$*,* $\varsigma {|}_{𝐕}$ *is transitive.*

**Definition 12.** *[*31*] A sequence* $\left\{v_{\text{n}}\right\}$ *in a MS* (𝐕,ϱ) *is semi-Cauchy if*


$$\begin{array}{c} \lim_{\text{n}\to \infty }\varrho (v_{\text{n}},v_{\text{n}+1})=0. \end{array}$$

Any Cauchy sequence is semi-Cauchy, while not being the opposing somewhat way.

**Lemma 1.** *[*29*] If* $\left\{v_{\text{n}}\right\}$ *retains a non-Cauchy sequence in a MS* (𝐕,ϱ)*, then* ∃ $\epsilon_{0}>0$ *and two subsequences* $\left\{v_{n_{j}}\right\}$ *and* $\left\{v_{l_{j}}\right\}$ *of* $\left\{v_{\text{n}}\right\}$ *verifying*

(i) $\text{j}\leq l_{j}<n_{j}\;\;\forall \;\text{j}\in \mathbb{N}$,
(ii) $\varrho (v_{l_{j}},v_{n_{j}})>\epsilon_{0}\;\;\forall \;\text{j}\in \mathbb{N}$,
(iii) $\varrho (v_{l_{j}},v_{{\text{n}}_{\text{j}-1}})\leq \epsilon_{0}\;\;\forall \;\text{j}\in \mathbb{N}$.*If* $\left\{v_{\text{n}}\right\}$ *is also semi-Cauchy, then*
(iv) $\lim_{\text{j}\to \infty }\varrho (v_{l_{j}},v_{n_{j}})=\epsilon_{0},$
(v) $\lim_{\text{j}\to \infty }\varrho (v_{l_{j}},v_{n_{j}+1})=\epsilon_{0},$
(vi) $\lim_{\text{j}\to \infty }\varrho (v_{l_{j}+1},v_{n_{j}})=\epsilon_{0},$
(vii) $\lim_{\text{j}\to \infty }\varrho (v_{l_{j}+1},v_{n_{j}+1})=\epsilon_{0}.$

**Proposition 3.** *For each* θ∈Θ *and* μ∈ℳ*,* (I) *and* (II) *remain identical, where*

(I) ϱ(ℏv,ℏw)≤θ(ϱ(v,w))+min{μ(ϱ(v,ℏv)),μ(ϱ(w,ℏw)),μ(ϱ(v,ℏw)),μ(ϱ(w,ℏv))},
  $$\begin{array}{c} \forall \;(v,w)\in \varsigma , \end{array}$$
(II) ϱ(ℏv,ℏw)≤θ(ϱ(v,w))+min{μ(ϱ(v,ℏv)),μ(ϱ(w,ℏw)),μ(ϱ(v,ℏw)),μ(ϱ(w,ℏv))},
  $$\begin{array}{c} \forall \;[v,w]\in \varsigma . \end{array}$$

*Proof.* The conclusion is determined through the use of the symmetry of ϱ. □

## Main results

In a moment, the subsequent aforementioned results are to be reported which guarantee the existence and uniqueness of fixed points of certain sorts of nonlinear almost contractions in relational MS.

**Theorem 3.** *Let* (𝐕,ϱ) *be a MS with a BR* ς*, while* ℏ:𝐕→𝐕 *a map. Also,*

(a) (𝐕,ϱ) *remains* ς*-complete,*
(b) ς *keeps up locally* ℏ*-transitive and* ℏ*-closed,*
(c) ∃ $v_{0}\in 𝐕$ *verifying* $(v_{0},\hbar v_{0})\in \varsigma$,
(d) ℏ *remains* ς*-continuous or* ς *retains* ϱ*-self-closed,*
(e) ∃ θ∈Θ *and* μ∈ℳ *with*
  $$\begin{array}{cc} \varrho (\hbar v,\hbar w) & \leq \theta (\varrho (v,w))+\min\{\mu (\varrho (v,\hbar w)),\mu (\varrho (w,\hbar v)),\mu (\varrho (v,\hbar v)),\mu (\varrho (w,\hbar w))\},\\ & \;\;\;\forall \;(v,w)\in \varsigma . \end{array}$$

*Then,* ℏ *encompasses a fixed point.*

*Proof.* To appeal to this conclusion, we shall execute the following steps.

**Step-1.** Beginning with $v_{0}\in 𝐕$, we construct the following sequence $\{v_{n}\}\subset 𝐕$:
  $$\begin{array}{c} v_{n}=\hbar^{n}(v_{0})=\hbar (v_{n-1}),\;\forall \;n\in \mathbb{N}. \end{array}$$ (2)
**Step-2.** Employing (a) and Proposition 2, we find
  $$\begin{array}{c} (\hbar^{n}v_{0},\hbar^{n+1}v_{0})\in \varsigma \end{array}$$
  which, in combination with (2), leads to
  $$\begin{array}{c} (v_{n},v_{n+1})\in \varsigma ,\;\forall \;n\in \mathbb{N}. \end{array}$$ (3)
**Step-3.** We shall reveal that $\left\{v_{n}\right\}$ is semi-Cauchy, i.e., $\lim_{n\to \infty }\varrho (v_{n},v_{n+1})=0$. Denoting $\varrho_{n}:=\varrho (v_{n},v_{n+1})$. If ∃ $n_{0}\in {\mathbb{N}}_{0}$ verifying $\varrho_{n_{0}}=\varrho (v_{n_{0}},v_{n_{0}+1})=0$, then through (2), we attain $\hbar (v_{n_{0}})=v_{n_{0}}$. Thus, $v_{n_{0}}$ remains a fixed point of ℏ. It indicates that we’re concluded.

In case $\varrho_{n}>0,\;\forall \;n\in {\mathbb{N}}_{0}$, by means of the contractive-condition (e) to (2) and (3), we turn up


$$\begin{array}{cc} \varrho (v_{n},v_{n+1}) & \leq \theta (\varrho_{n-1})+\min\{\mu (\varrho (v_{n-1},\hbar v_{n})),\mu (\varrho (v_{n},\hbar v_{n-1})),\mu (\varrho (v_{n-1},\hbar v_{n-1})),\\ & \;\;\mu (\varrho (v_{n},\hbar v_{n}))\}\\ & =\theta (\varrho_{n-1})+\min\{\mu (\varrho (v_{n-1},v_{n+1})),\mu (0),\mu (\varrho_{n-1}),\mu (\varrho_{n})\} \end{array}$$

so that


$$\begin{array}{c} \varrho_{n}\leq \theta (\varrho_{n-1}),\;\forall \;n\in \mathbb{N}. \end{array}$$ (4)

With the help of the attribute of θ in (4), we find


$$\begin{array}{c} \varrho_{n}\leq \theta (\varrho_{n-1})<\varrho_{n-1},\;\forall \;n\in \mathbb{N}. \end{array}$$

Thereby, $\left\{\varrho_{n}\right\}$ retains a decreasing sequence of positive reals, which is also bounded below by ‘0’; so ∃ R≥0 with


$$\begin{array}{c} \lim_{n\to \infty }\varrho_{n}=R. \end{array}$$ (5)

At this point, we contend that *R* = 0. If *R* > 0, then with (5), a premise of θ, and the resulting a limit in (4), we acquire


$$\begin{array}{c} R=\lim_{n\to \infty }\varrho_{n}\leq \lim_{n\to \infty }\theta (\varrho_{n-1})=\lim_{\varrho_{n}\to R^{+}}\theta (\varrho_{n-1})<R, \end{array}$$

This leads to *R* = 0 since it is ambiguous; thereby, we acquire


$$\begin{array}{c} \lim_{n\to \infty }\varrho_{n}=0. \end{array}$$ (6)

**Step-4.** We shall demonstrate that $\left\{v_{n}\right\}$ persists as Cauchy. In the reverse direction, if $\left\{v_{n}\right\}$ is not Cauchy, then from Lemma 1, ∃ $\epsilon_{0}>0$ and two subsequences $\left\{v_{n_{j}}\right\}$ and $\left\{v_{{\text{l}}_{j}}\right\}$ of $\left\{v_{n}\right\}$ that fulfill
  $$\begin{array}{c} j\leq {\text{l}}_{j}<n_{j},\;\varrho (v_{{\text{l}}_{j}},v_{n_{j}})>\epsilon_{0}\geq \varrho (v_{{\text{l}}_{j}},v_{n_{j-1}}),\;\forall \;j\in \mathbb{N}. \end{array}$$

Denoting $\delta_{j}:=\varrho (v_{{\text{l}}_{j}},v_{n_{j}})$. As $\left\{v_{n}\right\}$ is ς-preserving (by (3)) and $\left\{v_{n}\}\subset \hbar (𝐕\right)$ (by (2)), utilizing locally ℏ-transitivity property of ς, we conclude $(v_{{\text{l}}_{j}},v_{n_{j}})\in \varsigma$. Thus, through assumption (e), we derive


$$\begin{array}{cc} \varrho (v_{{\text{l}}_{j}+1},v_{n_{j}+1}) & =\varrho (\hbar v_{{\text{l}}_{j}},\hbar v_{n_{j}})\leq \theta (\varrho (v_{{\text{l}}_{j}},v_{n_{j}}))+\min\{\mu (\varrho (v_{{\text{l}}_{j}},\hbar v_{{\text{l}}_{j}})),\mu (\varrho (v_{n_{j}},\hbar v_{n_{j}})),\\ & \;\mu (\varrho (v_{{\text{l}}_{j}},\hbar v_{n_{j}})),\mu (\varrho (v_{n_{j}},\hbar v_{{\text{l}}_{j}}))\}, \end{array}$$

so that


$$\begin{array}{c} \varrho (v_{{\text{l}}_{j}+1},v_{n_{j}+1})\leq \theta (\delta_{j})+\min\{\mu (\varrho_{{\text{l}}_{j}}),\mu (\varrho_{n_{j}}),\mu (\varrho (v_{{\text{l}}_{j}},v_{n_{j}+1})),\mu (\varrho (v_{n_{j}},v_{{\text{l}}_{j}+1}))\}. \end{array}$$ (7)

Made with the limit in (7), Lemma (1), and the characterizations of θ and μ, we lead


$$\begin{array}{cc} \epsilon_{0} & =\lim_{j\to \infty }\varrho (v_{{\text{l}}_{j}+1},v_{n_{j}+1})\leq \lim_{j\to \infty }\theta (\delta_{j})+\min\{0,0,\lim_{j\to \infty }\mu (\varrho (v_{{\text{l}}_{j}},v_{n_{j}+1})),\lim_{j\to \infty }\mu (\varrho (v_{n_{j}},v_{{\text{l}}_{j}+1})))\}\\ & =\lim_{j\to \infty }\theta (\delta_{j})+0=\lim_{s\to \epsilon_{0}^{+}}\theta (s)<\epsilon_{0}, \end{array}$$

leading to contradict. It yields that $\left\{v_{n}\right\}$ is Cauchy. As $\left\{v_{n}\right\}$ is ς-preserving also, by ς-completeness of 𝐕, ∃ $v^{*}\in 𝐕$ enjoying $v_{n}\overset{\varrho }{⟶}v^{*}$.

**Step-5.** We shall employ the assumption (d) to confirm that $v^{*}\in Fix(\hbar )$. Made from (3) and $v_{n}\overset{\varrho }{⟶}v^{*}$, we pick up
  $$\begin{array}{c} v_{n+1}=\hbar (v_{n})\overset{\varrho }{⟶}\hbar (v^{*}). \end{array}$$

Employing uniqueness character of limit, we find $\hbar (v^{*})=v^{*}$. Hence, $v^{*}$ retains a fixed point of ℏ. □

**Theorem 4.** *Additionally to presumptions of Theorem 3, if* ℏ(𝐕) *is* $\varsigma^{s}$*-directed, then* ℏ *owns a unique fixed point.*

*Proof.* By Theorem 3, we conclude Fix(ℏ)≠∅. If we pick $v^{*},w^{*}\in Fix(\hbar )$, we acquire


$$\begin{array}{c} \hbar^{\text{n}}(v^{*})=v^{*}\;\text{and}\;\hbar^{\text{n}}(w^{*})=w^{*},\;\forall \;\text{n}\in {\mathbb{N}}_{0}. \end{array}$$ (8)

Since $v^{*},w^{*}\in \hbar (𝐕)$ and ℏ(𝐕) is $\varsigma^{s}$-directed, therefore ∃ $v_{0}\in 𝕍$ with $[v^{*},v_{0}]\in \varsigma$ and $[w^{*},v_{0}]\in \varsigma$. Define the aforementioned sequence:


$$\begin{array}{c} v_{\text{n}}=\hbar^{\text{n}}(v_{0})=\hbar (v_{\text{n}-1}),\;\forall \;\text{n}\in {\mathbb{N}}_{0}. \end{array}$$ (9)

Through (8), (9), assumption (a) and Proposition 2, we find


$$\begin{array}{c} {}[v^{*},v_{\text{n}}]\in \varsigma \;\text{and}\;[w^{*},v_{\text{n}}]\in \varsigma ,\;\forall \;\text{n}\in {\mathbb{N}}_{0}. \end{array}$$ (10)

Denoting $\mu_{n}:=\varrho (v^{*},v_{\text{n}})$. We shall derive that


$$\begin{array}{c} \lim_{n\to \infty }\mu_{n}=\lim_{n\to \infty }\varrho (v^{*},v_{\text{n}})=0. \end{array}$$ (11)

By (9), (10), contractive-condition (e) and Proposition 3, we attain


$$\begin{array}{cc} \varrho (v^{*},v_{\text{n}+1}) & =\varrho (\hbar v^{*},\hbar v_{\text{n}})\\ & \leq \theta (\varrho (v^{*},v_{\text{n}}))+\min\{\mu (\varrho (v^{*},\hbar v_{\text{n}})),\mu (\varrho (v_{\text{n}},\hbar v^{*})),\mu (\varrho (v^{*},\hbar v^{*})),\mu (\varrho (v_{\text{n}},\hbar v_{\text{n}}))\}\\ & =\theta (\varrho (v^{*},v_{\text{n}}))+\min\{\mu (\varrho (v^{*},v_{\text{n}})),\mu (\varrho (v_{\text{n}},v^{*})),0,\mu (\varrho (v_{\text{n}},v_{\text{n}+1})))\} \end{array}$$

so that


$$\begin{array}{c} \mu_{n+1}\leq \theta (\mu_{n}). \end{array}$$ (12)

If ∃ $n_{0}\in \mathbb{N}$ with $\mu_{n_{0}}=0$, then we come across $\hbar^{n_{0}}(v)=\hbar^{n_{0}}(\text{v})$ to conclude $\hbar^{n_{0}+1}(v)=\hbar^{n_{0}+1}(\text{v})$. This implies that $\mu_{n_{0}+1}=0$. Employing induction, we find $\mu_{n}=0,\;\forall \;n\geq n_{0},$ so that $\lim_{n\to \infty }\mu_{n}=0$. If $\mu_{n}>0,\;\forall \;n\in \mathbb{N}$, then utilizing the characteristic of θ, (12) deduces to $\mu_{n+1}<\mu_{n}$. Thereby, $\left\{\mu_{n}\right\}$ retains a decreasing sequence (of positive reals) with lower bound ‘0’; so ∃ $\overset{˘}{\mu }\geq 0$ with


$$\begin{array}{c} \lim_{n\to \infty }\mu_{n}=\overset{˘}{\mu }. \end{array}$$ (13)

At this point, we contend that $\overset{˘}{\mu }=0.$ If $\overset{˘}{\mu }>0$ then ensuing limit in (12), and through the use of (13), and the predicate of θ, we accomplish


$$\begin{array}{c} \overset{˘}{\mu }=\lim_{n\to \infty }\mu_{n}\leq \lim_{n\to \infty }\theta (\mu_{n-1})=\lim_{\mu_{n}\to {\overset{˘}{\mu }}^{+}}\theta (\mu_{n-1})<\overset{˘}{\mu }, \end{array}$$

which contradicting itself, leading to $\overset{˘}{\mu }=0$. This suggests that $\lim_{n\to \infty }\mu_{n}=0$. Accordingly, (11) is affirmed in every scenario. In similar fashion, we can ascertain that


$$\begin{array}{c} \lim_{n\to \infty }\varrho (w^{*},v_{\text{n}})=0. \end{array}$$ (14)

Through (11), (14) and the triangle inequality, we come across


$$\begin{array}{c} \varrho (v^{*},w^{*})\leq \varrho (v^{*},v_{\text{n}})+\varrho (v_{\text{n}},w^{*})\to 0\;\text{as}\;n\to \infty \end{array}$$

yielding $v^{*}=w^{*}$. This wraps up the proof.

The fixed point theorem of Alfuraidan et al. [25] is derived from Theorem 3 if BR ς is assumed to be the edge set of a given directed graph.

For universal BR $\varsigma ={𝐕}^{2}$, Theorem 4 deduces the following finding.

**Corollary 1.** *Let* (𝐕,ϱ) *be a complete MS and* ℏ:𝐕→𝐕 *a map. If* ∃ θ∈Θ *and* μ∈ℳ *that fulfills*


$$\begin{array}{c} \varrho (\hbar v,\hbar w)\leq \theta (\varrho (v,w))+\min\{\mu (\varrho (𝑣,\hbar w)),\mu (\varrho (w,\hbar v)),\mu (\varrho (𝑣,\hbar v)),\mu (\varrho (w,\hbar w))\},\;\forall \;v,w\in 𝐕, \end{array}$$

*then* ℏ *owns a unique fixed point.*

If we put μ(t)=0 in Corollary 1, we derive main finding of Lakshmikantham and Ćirić [30]. Taking θ(t)=c·t (0<c<1), we explore the primary insight of Turinici [31]. For θ(t)=c·t (0<c<1), and μ(t)=a·t (a≥0), Corollary 1 reduces to Theorem 2 (i.e., the fixed point theorem of Babu et al. [10]).

## Illustrative examples

The intended effect of this aspect is to offer three instances which reinforce concepts outlined in the previous section.

**Example 1.** *Let* 𝐕=[0,1] *with Euclidean metric* ϱ *and BR* ς:=≥*. Then,* (𝐕,ϱ) *is* ς*-complete MS. Let the map* ℏ:𝐕→𝐕 *be defined by*


$$\begin{array}{c} \hbar (v)=\left\{ \begin{array}{cc} 0, & \text{if}\;v=1\\ 2/3, & \text{otherwise}. \end{array}\right. \end{array}$$

*Then,* ς *is* ℏ*-closed and* ℏ *is* ς*-continuous.*

*Define the functions* θ(𝑡)=2𝑡/3 *and* μ(𝑡)=3𝑡/4*. Then* θ∈Θ *and* μ∈ℳ*. For each* (v,w)∈ς*, we attain*


$$\begin{array}{c} \varrho (\hbar v,\hbar w)\leq \theta (\varrho (v,w))+\min\{\mu (\varrho (𝑣,\hbar w)),\mu (\varrho (w,\hbar v)),\mu (\varrho (𝑣,\hbar v)),\mu (\varrho (w,\hbar w))\}, \end{array}$$

*i.e.,* ℏ *verifies the contraction-inequality (e) of Theorem 3. Thus, all the hypotheses of Theorem 3 are fulfilled. In a comparable vein, we can validate each of the premises of Theorem 4. Thereby,* ℏ *owns a fixed point leading to* Fix(ℏ)={2/3}.

**Example 2.** *Let* 𝐕=(0,4] *with Euclidean metric* ϱ(v,w)=|v−w|*. Take a BR* ς=[1,2]∪[3,4] *on* 𝐕*. Thereby,* (𝐕,ϱ) *is* ς*-complete MS. Let the map* ℏ:𝐕→𝐕 *be defined by*


$$\begin{array}{c} \hbar (v)=\left\{ \begin{array}{c} 1,\;if\;0\leq v<3\\ 4,\;if\;3\leq v\leq 4. \end{array}\right. \end{array}$$

*Then,* ℏ *is* ς*-continuous and* ς *is* ℏ*-closed. Moreover,* ℏ *fulfills the contraction-inequality (e) for any arbitrary* θ∈Θ *and* μ(𝑡)=2𝑡*. Thus, by Theorem 3,* ℏ *owns a fixed point. Indeed, here* ℏ *encompasses two fixed points:* $v^{*}=1$ *and* $w^{*}=4$.

*Now,* ℏ(𝐕)={1,4} *is not* ς*-directed set as* ∄ v∈𝐕 *for which* (1,v)∈ς *and* (4,v)∈ς *hold simultaneously. Hence, Theorem 4, which confirms the uniqueness of fixed points, is not effective in this instance.*

**Example 3.** *Consider* 𝐕=[0,2] *with Euclidean metric* ϱ*. Define a map* ℏ:𝐕→𝐕 *by*


$$\begin{array}{c} \hbar (v)=\left\{ \begin{array}{c} v/2,\;if\;0\leq v<1\\ 2,\;\;if\;1\leq v\leq 2. \end{array}\right. \end{array}$$

*Define a BR* ς={(0,1),(0,2)} *on* 𝐕*. Clearly,* (𝐕,ϱ) *is* ς*-complete MS. Also,* ς *forms a locally* ℏ*-transitive,* ℏ*-closed and* ϱ*-self-closed BR. Moreover,* ℏ *fulfills the contractivity condition* (e) *for the auxiliary functions* θ(𝑡)=𝑡/2 *and* μ(𝑡)=3𝑡*. All the other requirements of Theorem 3 are also met.*

*Since there isn’t an element in* 𝐕 *that is at the same time* ς*-comparative with* ℏ(4/5)=2/5 *and* ℏ(1)=2, ℏ(𝐕) *is not* $\varsigma^{s}$*-directed. It turns out that the present example fails to satisfy Theorem 4. Indeed,* ℏ *admits two fixed points:* $v^{*}=0$ *and* $w^{*}=2$.

**Remark 1.** *Example 3 cannot be covered by the main results of Babu et al. [*10*] for if we take* v=0 *and* v=1*, then the contraction condition of Babu et al. [*10*] becomes*


$$\begin{array}{c} 2\leq 𝑐\mathrm{.1}+𝑎.\min\{0,1,2,1\}, \end{array}$$

*which contradicts to* 0<𝑐<1*. Similarly, the contraction condition of Turinici* [31] *is also never satisfied. Moreover, BR* ς *is irreflexive, therefore above example is not covered by the outcomes of Alfuraidan et al.* [25]*. This substantiates the utility and novelty of Theorems 3 and 4 over the corresponding results of Babu et al.* [10]*, Alfuraidan et al.* [25]*, and Turinici* [31].

## Applications To BVP

Let’s examine the subsequent BVP:


$$\begin{array}{c} \left\{ \begin{array}{c} \vartheta^{\prime }(\iota )=ϝ(\iota ,\vartheta (\iota )),\;\iota \in [0,l]\\ \vartheta (0)=\vartheta (l) \end{array}\right. \end{array}$$ (15)

whereas ϝ:[0,l]×ℝ→ℝ retains continuous.

In the aftermath, Λ will denote the family of increasing functions $\theta :{\mathbb{R}}^{+}\to {\mathbb{R}}^{+}$ verifying $\theta (\text{t})<\text{t},\;\forall \;\text{t}\in {\mathbb{R}}^{+}⧵\{0\}$ and $\lim_{r\to {\text{t}}^{+}}\theta (\text{r})<\text{t},\;\forall \;\text{t}\in {\mathbb{R}}^{+}⧵\{0\}$. Obviously, Λ⊂ϝ.

A function $\tilde{\vartheta }\in {𝒞}^{\prime }[0,l]$ is termed as a lower solution of (15) if


$$\begin{array}{c} \left\{ \begin{array}{c} {\tilde{\vartheta }}^{\prime }(\iota )\leq ϝ(\iota ,\tilde{\vartheta }(\iota )),\;\iota \in [0,l]\\ \tilde{\vartheta }(0)\leq \tilde{\vartheta }(l). \end{array}\right. \end{array}$$

Also, a function $\overset{ˇ}{\vartheta }\in {𝒞}^{\prime }[0,l]$ is termed as an upper solution of (15) if


$$\begin{array}{c} \left\{ \begin{array}{c} {\overset{ˇ}{\vartheta }}^{\prime }(\iota )\geq ϝ(\iota ,\overset{ˇ}{\vartheta }(\iota )),\;\iota \in [0,l]\\ \overset{ˇ}{\vartheta }(0)\geq \overset{ˇ}{\vartheta }(l). \end{array}\right. \end{array}$$

The central findings of this section are, like earlier outlined:

**Theorem 5.** *Alongside the Problem (15), if* ∃ β>0*, and* θ∈Λ *that fulfill*


$$\begin{array}{c} 0\leq [ϝ(\iota ,b)+\beta b]-[ϝ(\iota ,a)+\beta a]\leq \beta \theta (b-a),\;\forall \;\iota \in [0,l]\;\text{and}\;a,b\in \mathbb{R}\;\text{with}\;a\leq b, \end{array}$$ (16)

*then Problem (15) owns a unique solution when it possesses a lower solution.*

*Proof.* It is possible to reformulate Eq. (15) as


$$\begin{array}{c} \left\{ \begin{array}{c} \vartheta^{\prime }(\iota )+\beta \vartheta (\iota )=ϝ(\iota ,\vartheta (\iota ))+\beta \vartheta (\iota ),\;\forall \;\iota \in [0,l]\\ \vartheta (0)=\vartheta (l). \end{array}\right. \end{array}$$

The Fredholm integral equation identical to the above BVP is


$$\begin{array}{c} \vartheta (\iota )=\int_{0}^{l}\Gamma (\iota ,\theta )[ϝ(\theta ,\vartheta (\theta ))+\beta \vartheta (\theta )]d\theta , \end{array}$$ (17)

for which the Green function Γ(ι,θ) is


$$\begin{array}{c} \Gamma (\iota ,\theta )=\left\{ \begin{array}{c} \frac{e^{\beta (l+\theta -\iota )}}{e^{\beta l}-1},\;0\leq \theta <\iota \leq l\\ \frac{e^{\beta (\theta -\iota )}}{e^{\beta l}-1},\;0\leq \iota <\theta \leq l. \end{array}\right. \end{array}$$

Obviously, Γ remains continuous function of ι and θ.

Denoting 𝐕:=𝒞[0,l]. Define a map ℏ:𝐕→𝐕 by


$$\begin{array}{c} (\hbar \vartheta )(\iota )=\int_{0}^{l}\Gamma (\iota ,\theta )[ϝ(\theta ,\vartheta (\theta ))+\beta \vartheta (\theta )]d\theta ,\;\forall \;\iota \in [0,l]. \end{array}$$ (18)

Construct a BR ς on 𝐕 as below:


$$\begin{array}{c} \varsigma =\{(\vartheta ,\omega )\in 𝐕\times 𝐕:\vartheta (\iota )\leq \omega (\iota ),\;\forall \;\iota \in [0,l]\}. \end{array}$$ (19)

Induce a metric ϱ on 𝐕 such that


$$\begin{array}{c} \varrho (\vartheta ,\omega )=\underset{\iota \in [0,l]}{\sup}|\vartheta (\iota )-\omega (\iota )|,\;\forall \;\vartheta ,\omega \in 𝐕. \end{array}$$ (20)

We now confirm every premise of Theorems 3 and 4.

(a) As MS (𝐕,ϱ) remains complete, it must be ς-complete,
(b) Let $\tilde{\vartheta }\in {𝒞}^{\prime }[0,l]$ be a lower solution of (15). Then, we achieve
  $$\begin{array}{c} {\tilde{\vartheta }}^{\prime }(\iota )+\beta \tilde{\vartheta }(\iota )\leq ϝ(\iota ,\tilde{\vartheta }(\iota ))+\beta \tilde{\vartheta }(\iota ),\;\forall \;\iota \in [0,l]. \end{array}$$

Having taken multiplication to $e^{\beta \iota }$, we receive


$$\begin{array}{c} (\tilde{\vartheta }(\iota )e^{\beta \iota }{)}^{\prime }\leq [ϝ(\iota ,\tilde{\vartheta }(\iota ))+\beta \tilde{\vartheta }(\iota )]e^{\beta \iota },\;\forall \;\iota \in [0,l], \end{array}$$

so that


$$\begin{array}{c} \tilde{\vartheta }(\iota )e^{\beta \iota }\leq \tilde{\vartheta }(0)+\int_{0}^{\iota }[ϝ(\theta ,\tilde{\vartheta }(\theta ))+\beta \tilde{\vartheta }(\theta )]e^{\beta \theta }d\theta ,\;\forall \;\iota \in [0,l]. \end{array}$$ (21)

From $\tilde{\vartheta }(0)\leq \tilde{\vartheta }(l)$, we determine


$$\begin{array}{c} \tilde{\vartheta }(0)e^{\beta l}\leq \tilde{\vartheta }(l)e^{\beta l}\leq \tilde{\vartheta }(0)+\int_{0}^{l}[ϝ(\theta ,\tilde{\vartheta }(\theta ))+\beta \tilde{\vartheta }(\theta )]e^{\beta \theta }d\theta , \end{array}$$

implying thereby


$$\begin{array}{c} \tilde{\vartheta }(0)\leq \int_{0}^{l}\frac{e^{\beta \theta }}{e^{\beta l}-1}[ϝ(\theta ,\tilde{\vartheta }(\theta ))+\beta \tilde{\vartheta }(\theta )]d\theta . \end{array}$$ (22)

Owing to (21) and (22), we find


$$\begin{array}{cc} \tilde{\vartheta }(\iota )e^{\beta \iota } & \leq \int_{0}^{l}\frac{e^{\beta \theta }}{e^{\beta l}-1}[ϝ(\theta ,\tilde{\vartheta }(\theta ))+\beta \tilde{\vartheta }(\theta )]d\theta +\int_{0}^{\iota }e^{\beta \theta }[ϝ(\theta ,\tilde{\vartheta }(\theta ))+\beta \tilde{\vartheta }(\theta )]d\theta \\ & =\int_{0}^{\iota }\frac{e^{\beta (l+\theta )}}{e^{\beta l}-1}[ϝ(\theta ,\tilde{\vartheta }(\theta ))+\beta \tilde{\vartheta }(\theta )]d\theta +\int_{\iota }^{l}\frac{e^{\beta \theta }}{e^{\beta l}-1}[ϝ(\theta ,\tilde{\vartheta }(\theta ))+\beta \tilde{\vartheta }(\theta )]d\theta , \end{array}$$

implying thereby


$$\begin{array}{cc} \tilde{\vartheta }(\iota ) & \leq \int_{0}^{\iota }\frac{e^{\beta (l+\theta -\iota )}}{e^{\beta l}-1}[ϝ(\theta ,\tilde{\vartheta }(\theta ))+\beta \tilde{\vartheta }(\theta )]d\theta +\int_{\iota }^{l}\frac{e^{\beta (\theta -\iota )}}{e^{\beta l}-1}[ϝ(\theta ,\tilde{\vartheta }(\theta ))+\beta \tilde{\vartheta }(\theta )]d\theta \\ & =\int_{0}^{l}\Gamma (\iota ,\theta )[ϝ(\theta ,\tilde{\vartheta }(\theta ))+\beta \tilde{\vartheta }(\theta )]d\theta \\ & =(\hbar \tilde{\vartheta })(\iota ),\;\forall \;\iota \in [0,l], \end{array}$$

so that $(\tilde{\vartheta },\hbar \tilde{\vartheta })\in \varsigma$.

(c) Obviously ς is transitive, it retains locally ℏ-transitive BR. To prove the ℏ-closedness of ς, let ϑ,ω∈𝐕 verifying (ϑ,ω)∈ς. By (16), we attain
  $$\begin{array}{c} ϝ(\iota ,\vartheta (\iota ))+\beta \vartheta (\iota )\leq ϝ(\iota ,\omega (\iota ))+\beta \omega (\iota ),\;\forall \;\iota \in [0,l]. \end{array}$$ (23)

From (18), (23) and Γ(ι,θ)>0, for all ι,θ∈[0,l], we conclude


$$\begin{array}{cc} \left(\hbar \vartheta )(\iota \right) & =\int_{0}^{l}\Gamma (\iota ,\theta )[ϝ(\theta ,\vartheta (\theta ))+\beta \vartheta (\theta )]d\theta \\ & \leq \int_{0}^{l}\Gamma (\iota ,\theta )[ϝ(\theta ,\omega (\theta ))+\beta \omega (\theta )]d\theta \\ & =(\hbar \omega )(\iota ),\;\text{for all}\;\iota \in [0,l], \end{array}$$

which along with (19) concludes that (ℏϑ,ℏω)∈ς. Thereby, ς remains ℏ-closed.

(d) As Γ and ϝ both are continuous, ℏ is continuous and hence ς-continuous.
(e) Let ϑ,ω∈𝐕 verifying (ϑ,ω)∈ς. Owing to (16), (18) and (20), we attain
  $$\begin{array}{cc} \;\varrho (\hbar \vartheta ,\hbar \omega ) & =\underset{\iota \in [0,l]}{\sup}|(\hbar \vartheta )(\iota )-(\hbar \omega )(\iota )|=\underset{\iota \in [0,l]}{\sup}((\hbar \omega )(\iota )-(\hbar \vartheta )(\iota ))\\ & \leq \underset{\iota \in [0,l]}{\sup}\int_{0}^{l}\Gamma (\iota ,\theta )[ϝ(\theta ,\omega (\theta ))+\beta \omega (\theta )-ϝ(\theta ,\vartheta (\theta ))-\beta \vartheta (\theta )]d\theta \\ & \leq \underset{\iota \in [0,l]}{\sup}\int_{0}^{l}\Gamma (\iota ,\theta )\beta \theta (\omega (\theta )-\vartheta (\theta ))d\theta . \end{array}$$ (24)

As 0≤ω(θ)−ϑ(θ)≤ϱ(ϑ,ω) and θ is increasing, we attain


$$\begin{array}{c} \theta (\omega (\theta )-\vartheta (\theta ))\leq \theta (\varrho (\vartheta ,\omega )). \end{array}$$

From last inequality, (24) reduces to


$$\begin{array}{cc} \varrho (\hbar \vartheta ,\hbar \omega ) & \leq \beta \theta (\varrho (\vartheta ,\omega ))\underset{\iota \in [0,l]}{\sup}\int_{0}^{l}\Gamma (\iota ,\theta )d\theta \\ & =\beta \theta (\varrho (\vartheta ,\omega ))\underset{\iota \in [0,l]}{\sup}\frac{1}{e^{\beta l}-1}[\frac{1}{\beta }e^{\beta (l+\theta -\iota )}{|}_{0}^{\iota }+\frac{1}{\beta }e^{\beta (\theta -\iota )}{|}_{\iota }^{l}]\\ & =\beta \theta (\varrho (\vartheta ,\omega ))\frac{1}{\beta (e^{\beta l}-1)}(e^{\beta l}-1)\\ & =\theta (\varrho (\vartheta ,\omega )), \end{array}$$

so that


$$\begin{array}{cc} & \varrho (\hbar \vartheta ,\hbar \omega )\leq \theta (\varrho (\vartheta ,\omega ))+\min\{\vartheta (\varrho (\vartheta ,\hbar \vartheta )),\vartheta (\varrho (\omega ,\hbar \omega )),\vartheta (\varrho (\vartheta ,\hbar \omega )),\vartheta (\varrho (\omega ,\hbar \vartheta ))\},\\ & \;\;\;\;\;\forall \;\vartheta ,\omega \in 𝐕\;with\;(\vartheta ,\omega )\in \varsigma \end{array}$$

where ϑ∈Λ is arbitrary.

Thus in all, the suppositions (a)−(e) of Theorem 3 hold; so ℏ owns a fixed point.

Choose ϑ,ω∈𝐕 arbitrarily yielding thereby ℏ(ϑ),ℏ(ω)∈ℏ(𝐕). Set ν:=max{ℏϑ,ℏω} implying thereby (ℏϑ,ν)∈ς and (ℏω,ν)∈ς. Hence, ℏ(𝐕) forms $\varsigma^{s}$-directed set. Thus, through Theorem 4, ℏ enjoys a unique fixed point, that turns out the unique solution of Problem (15). □

**Theorem 6.** *Alongside the Problem (15), if* ∃ β>0*, and* θ∈Λ *that fulfill*


$$\begin{array}{c} 0\leq [ϝ(\iota ,b)+\beta b]-[ϝ(\iota ,a)+\beta a]\leq \beta \theta (b-a),\;\forall \;\iota \in [0,l]\;\text{and}\;a,b\in \mathbb{R}\;\text{with}\;a\leq b, \end{array}$$ (25)

*then Problem (15) owns a unique solution when it possesses an upper solution.*

*Proof.* Take 𝐕:=𝒞[0,l] with a metric ϱ and a map ℏ:𝐕→𝐕 to Theorem 5. On 𝐕, induce a BR ς as below:


$$\begin{array}{c} \varsigma^{\prime }=\{(\vartheta ,\omega )\in 𝐕\times 𝐕:\vartheta (\iota )\geq \omega (\iota ),\;\forall \;\iota \in [0,l]\}. \end{array}$$ (26)

We now confirm every premise of Theorems 3 and 4.

(a) As MS (𝐕,ϱ) remains complete, it must be $\varsigma^{\prime }$-complete,
(b) Let $\overset{ˇ}{\vartheta }\in {𝒞}^{\prime }[0,l]$ be a upper solution of (15). Then, we achieve

$$\begin{array}{c} {\overset{ˇ}{\vartheta }}^{\prime }(\iota )+\beta \overset{ˇ}{\vartheta }(\iota )\geq ϝ(\iota ,\overset{ˇ}{\vartheta }(\iota ))+\beta \overset{ˇ}{\vartheta }(\iota ),\;\forall \;\iota \in [0,l]. \end{array}$$

Having taken multiplication to $e^{\beta \iota }$, we receive


$$\begin{array}{c} (\overset{ˇ}{\vartheta }(\iota )e^{\beta \iota }{)}^{\prime }\geq [ϝ(\iota ,\overset{ˇ}{\vartheta }(\iota ))+\beta \overset{ˇ}{\vartheta }(\iota )]e^{\beta \iota },\;\forall \;\iota \in [0,l], \end{array}$$

so that


$$\begin{array}{c} \overset{ˇ}{\vartheta }(\iota )e^{\beta \iota }\geq \overset{ˇ}{\vartheta }(0)+\int_{0}^{\iota }[ϝ(\theta ,\overset{ˇ}{\vartheta }(\theta ))+\beta \overset{ˇ}{\vartheta }(\theta )]e^{\beta \theta }d\theta ,\;\forall \;\iota \in [0,l]. \end{array}$$ (27)

As $\overset{ˇ}{\vartheta }(0)\geq \overset{ˇ}{\vartheta }(l)$, we attain


$$\begin{array}{c} \overset{ˇ}{\vartheta }(0)e^{\beta l}\geq \overset{ˇ}{\vartheta }(l)e^{\beta l}\geq \overset{ˇ}{\vartheta }(0)+\int_{0}^{l}[ϝ(\theta ,\overset{ˇ}{\vartheta }(\theta ))+\beta \overset{ˇ}{\vartheta }(\theta )]e^{\beta \theta }d\theta , \end{array}$$

implying thereby


$$\begin{array}{c} \overset{ˇ}{\vartheta }(0)\geq \int_{0}^{l}\frac{e^{\beta \theta }}{e^{\beta l}-1}[ϝ(\theta ,\overset{ˇ}{\vartheta }(\theta ))+\beta \overset{ˇ}{\vartheta }(\theta )]d\theta . \end{array}$$ (28)

Owing to (27) and (28), we find


$$\begin{array}{cc} \overset{ˇ}{\vartheta }(\iota )e^{\beta \iota } & \geq \int_{0}^{l}\frac{e^{\beta \theta }}{e^{\beta l}-1}[ϝ(\theta ,\overset{ˇ}{\vartheta }(\theta ))+\beta \overset{ˇ}{\vartheta }(\theta )]d\theta +\int_{0}^{\iota }e^{\beta \theta }[ϝ(\theta ,\overset{ˇ}{\vartheta }(\theta ))+\beta \overset{ˇ}{\vartheta }(\theta )]d\theta \\ & =\int_{0}^{\iota }\frac{e^{\beta (l+\theta )}}{e^{\beta l}-1}[ϝ(\theta ,\overset{ˇ}{\vartheta }(\theta ))+\beta \overset{ˇ}{\vartheta }(\theta )]d\theta +\int_{\iota }^{l}\frac{e^{\beta \theta }}{e^{\beta l}-1}[ϝ(\theta ,\overset{ˇ}{\vartheta }(\theta ))+\beta \overset{ˇ}{\vartheta }(\theta )]d\theta , \end{array}$$

implying thereby


$$\begin{array}{cc} \overset{ˇ}{\vartheta }(\iota ) & \geq \int_{0}^{\iota }\frac{e^{\beta (l+\theta -\iota )}}{e^{\beta l}-1}[ϝ(\theta ,\overset{ˇ}{\vartheta }(\theta ))+\beta \overset{ˇ}{\vartheta }(\theta )]d\theta +\int_{\iota }^{l}\frac{e^{\beta (\theta -\iota )}}{e^{\beta l}-1}[ϝ(\theta ,\overset{ˇ}{\vartheta }(\theta ))+\beta \overset{ˇ}{\vartheta }(\theta )]d\theta \\ & =\int_{0}^{l}\Gamma (\iota ,\theta )[ϝ(\theta ,\overset{ˇ}{\vartheta }(\theta ))+\beta \overset{ˇ}{\vartheta }(\theta )]d\theta \\ & =(\hbar \overset{ˇ}{\vartheta })(\iota ),\;\forall \;\iota \in [0,l], \end{array}$$

so that $(\overset{ˇ}{\vartheta },\hbar \overset{ˇ}{\vartheta })\in \varsigma^{\prime }$.

(c) Obviously $\varsigma^{\prime }$ is transitive, it retains locally ℏ-transitive BR. To prove the ℏ-closedness of $\varsigma^{\prime }$, let ϑ,ω∈𝐕 verifying $(\vartheta ,\omega )\in \varsigma^{\prime }$. By (25), we attain
  $$\begin{array}{c} ϝ(\iota ,\vartheta (\iota ))+\beta \vartheta (\iota )\geq ϝ(\iota ,\omega (\iota ))+\beta \omega (\iota ),\;\text{for all}\;\iota \in [0,l]. \end{array}$$ (29)

From (18), (29) and Γ(ι,θ)>0, for all ι,θ∈[0,l], we conclude


$$\begin{array}{cc} \left(\hbar \vartheta )(\iota \right) & =\int_{0}^{l}\Gamma (\iota ,\theta )[ϝ(\theta ,\vartheta (\theta ))+\beta \vartheta (\theta )]d\theta \\ & \geq \int_{0}^{l}\Gamma (\iota ,\theta )[ϝ(\theta ,\omega (\theta ))+\beta \omega (\theta )]d\theta \\ & =(\hbar \omega )(\iota ),\;\text{for all}\;\iota \in [0,l], \end{array}$$

which along with (26) concludes that $(\hbar \vartheta ,\hbar \omega )\in \varsigma^{\prime }$. Thereby, $\varsigma^{\prime }$ remains ℏ-closed.

(d) As Γ and ϝ both are continuous, ℏ is continuous and hence $\varsigma^{\prime }$-continuous.
(e) Let ϑ,ω∈𝐕 verifying $(\vartheta ,\omega )\in \varsigma^{\prime }$. Owing to (25), (18) and (20), we attain
  $$\begin{array}{cc} \;\varrho (\hbar \vartheta ,\hbar \omega ) & =\underset{\iota \in [0,l]}{\sup}|(\hbar \vartheta )(\iota )-(\hbar \omega )(\iota )|=\underset{\iota \in [0,l]}{\sup}((\hbar \omega )(\iota )-(\hbar \vartheta )(\iota ))\\ & \leq \underset{\iota \in [0,l]}{\sup}\int_{0}^{l}\Gamma (\iota ,\theta )[ϝ(\theta ,\omega (\theta ))+\beta \omega (\theta )-ϝ(\theta ,\vartheta (\theta ))-\beta \vartheta (\theta )]d\theta \\ & \leq \underset{\iota \in [0,l]}{\sup}\int_{0}^{l}\Gamma (\iota ,\theta )\beta \theta (\omega (\theta )-\vartheta (\theta ))d\theta . \end{array}$$ (30)

As 0≤ϑ(θ)−ω(θ)≤ϱ(ϑ,ω) and θ is increasing, we attain


$$\begin{array}{c} \theta (\omega (\theta )-\vartheta (\theta ))\leq \theta (\varrho (\vartheta ,\omega )). \end{array}$$

From last inequality, (30) reduces to


$$\begin{array}{cc} \varrho (\hbar \vartheta ,\hbar \omega ) & \leq \beta \theta (\varrho (\vartheta ,\omega ))\underset{\iota \in [0,l]}{\sup}\int_{0}^{l}\Gamma (\iota ,\theta )d\theta \\ & =\beta \theta (\varrho (\vartheta ,\omega ))\underset{\iota \in [0,l]}{\sup}\frac{1}{e^{\beta l}-1}[\frac{1}{\beta }e^{\beta (l+\theta -\iota )}{|}_{0}^{\iota }+\frac{1}{\beta }e^{\beta (\theta -\iota )}{|}_{\iota }^{l}]\\ & =\beta \theta (\varrho (\vartheta ,\omega ))\frac{1}{\beta (e^{\beta l}-1)}(e^{\beta l}-1)\\ & =\theta (\varrho (\vartheta ,\omega )), \end{array}$$

so that


$$\begin{array}{cc} & \varrho (\hbar \vartheta ,\hbar \omega )\leq \theta (\varrho (\vartheta ,\omega ))+\min\{\vartheta (\varrho (\vartheta ,\hbar \vartheta )),\vartheta (\varrho (\omega ,\hbar \omega )),\vartheta (\varrho (\vartheta ,\hbar \omega )),\vartheta (\varrho (\omega ,\hbar \vartheta ))\},\\ & \;\;\;\;\;\forall \;\vartheta ,\omega \in 𝐕\;with\;(\vartheta ,\omega )\in \varsigma^{\prime } \end{array}$$

where ϑ∈Λ is arbitrary.

Thus in all, the suppositions (a)−(e) of Theorem 3 hold; so ℏ owns a fixed point.

Choose ϑ,ω∈𝐕 arbitrarily yielding thereby ℏ(ϑ),ℏ(ω)∈ℏ(𝐕). Set ν:=min{ℏϑ,ℏω} implying thereby $(\hbar \vartheta ,\nu )\in \varsigma^{\prime }$ and $(\hbar \omega ,\nu )\in \varsigma^{\prime }$. Hence, ℏ(𝐕) forms $\varsigma^{\prime s}$-directed set. Thus, through Theorem 4, ℏ enjoys a unique fixed point, that turns out the unique solution of Problem (15).

We evaluate the following numerical example for the purpose to demonstrate Theorem 5.

**Example 4.** *Let* ϝ(ι,ϑ(ι))=cosι *for* 0≤ι≤π*. Then* ϝ *is a continuous function. Herein,* $\bar{\vartheta }=0$ *serves as a lower solution to* $\vartheta^{\prime }(\iota )=\cos\iota$*. So far, Theorem 5 can be employed for the given problem; consequently,* ϑ(ι)=sinι *remains the unique solution.*

## Conclusions

This paper addressed fixed point insights on an MS with a locally ℏ-transitive BR with referred to to a nonlinear articulate of almost contractions. Certain instances are also conducted to convey our findings. We also deduced a nonlinear formalism of typical fixed point finding of Babu et al. [10] throughout the study, owing to the constraint $\varsigma ={𝐕}^{2}$. Fortunately our findings allow us to generate various latest fixed point results, especially those reported from Lakshmikantham and Ćirić [30], Turinici [31], and Alfuraidan et al. [25]. The investigation clarifies that the concepts presented here can be implemented to solve nonlinear integral equations in the presence of either an upper or a lower solution.

Recognizing the critical significance of the relation-theoretic fixed point paradigm, we identify the subsequent prospects for upcoming studies:

1. optimizing the attributes of gauge functions θ and μ;
2. for enhancing our insights across fuzzy MS (on the lines of Zahid et al. [34]) and *b*-MS (on the lines of Zada et al. [35]);
3. extending our outcomes by examining the common fixed point theorems for two maps;
4. incorporating our findings to nonlinear integral equations (on the lines of Shoaib et al. [36], and Xu et al. [37]) and functional equations (on the lines of Monica and Kumar [38]), Sarwar et al. [39], and Shoaib et al. [40]).

### Abbreviations

**Table:** 

ℝ: Set of real numbers
ℕ: Set of natural numbers
${\mathbb{N}}_{0}:=\mathbb{N}\cup \{0\}$
${\mathbb{R}}^{+}:=[0,\infty )$
BCP: Banach contraction principle
BR: binary relation
BVP: boundary value problem
MS: metric space
𝒞(𝒥): Space of real continuous functions in interval 𝒥
${𝒞}^{\prime }(𝒥)$: Space of real continuously differentiable functions in interval 𝒥
Fix(ℏ): Set of fixed points of a self-map ℏ.
